# Severe acute respiratory syndrome coronavirus‐2 natural animal reservoirs and experimental models: systematic review

**DOI:** 10.1002/rmv.2196

**Published:** 2020-11-18

**Authors:** Salma Younes, Nadin Younes, Farah Shurrab, Gheyath K. Nasrallah

**Affiliations:** ^1^ Biomedical Research Center Member of QU Health Qatar University Doha Qatar; ^2^ Department of Biomedical Science College of Health Sciences Member of QU Health Qatar University Doha Qatar

**Keywords:** animal hosts, animal models, Covid‐19, SARS‐CoV‐2, therapeutic agents, vaccines

## Abstract

The current severe acute respiratory syndrome coronavirus‐2 (SARS‐CoV‐2) outbreak has been rapidly spreading worldwide, causing serious global concern. The role that animal hosts play in disease transmission is still understudied and researchers wish to find suitable animal models for fundamental research and drug discovery. In this systematic review, we aimed to compile and discuss all articles that describe experimental or natural infections with SARS‐CoV‐2, from the initial discovery of the virus in December 2019 through to October 2020. We systematically searched four databases (Scopus, PubMed, Science Direct and Web of Science). The following data were extracted from the included studies: type of infection (natural or experimental), age, sample numbers, dose, route of inoculation, viral replication, detection method, clinical symptoms and transmission. Fifty‐four studies were included, of which 34 were conducted on animal reservoirs (naturally or experimentally infected), and 20 involved models for testing vaccines and therapeutics. Our search revealed that *Rousettus aegyptiacus* (fruit bats), pangolins, felines, mink, ferrets and rabbits were all susceptible to SARS‐CoV‐2, while dogs were weakly susceptible and pigs, poultry, and tree shrews were not. In addition, virus replication in mice, mink, hamsters and ferrets resembled subclinical human infection, so these animals might serve as useful models for future studies to evaluate vaccines or antiviral agents and to study host‐pathogen interactions. Our review comprehensively summarized current evidence on SARS‐CoV‐2 infection in animals and their usefulness as models for studying vaccines and antiviral drugs. Our findings may direct future studies for vaccine development, antiviral drugs and therapeutic agents to manage SARS‐CoV‐2‐caused diseases.

**Abbreviations**
ACE2: angiotensin‐converting enzyme 2
Covid‐19: coronavirus disease 2019
CoVs: coronaviruses; CRISPR, clustered Regularly Interspaced Short Palindromic Repeats Repetitive; ELISA, enzyme‐linked immunosorbent assay; HCQ, hydroxychloroquine sulphate; MERS‐CoV, middle east respiratory syndrome coronavirus; MESH, medical subject headings
NHPs: non‐human primates
NAbs: neutralizing antibodies
PRISMA: preferred reporting items for systematic reviews and meta‐analyses
RBD: receptor binding domain
SARS‐CoV‐2: severe acute respiratory syndrome coronavirus 2
URT: upper respiratory tract

## 1 INTRODUCTION

The outbreak of coronavirus disease 2019 (Covid‐19) caused by the novel severe acute respiratory syndrome coronavirus 2 (SARS‐CoV‐2), has triggered a serious global public health problem and is spreading in almost all countries around the globe with 45,797,600 confirmed cases and 1,191,670 mortalities, as of 30 October 2020.^1^ The current outbreak of SARS‐CoV‐2 is the third outbreak of animal coronavirus transmission to humans in the past 2 decades.

SARS‐CoV‐2 belongs to the coronaviruses (CoVs), a genus in the *Coronaviridae* family. Most known CoVs infect animals, are associated with self‐limiting diseases and have a narrow host range.^2^ However, some zoonotic CoVs have a high capability to mutate, which enables the virus to cross the species barrier to infect humans, such as SARS‐CoV, middle east respiratory syndrome coronavirus (MERS‐CoV), and SARS‐CoV‐2 zoonotic viruses, which have caused severe acute respiratory diseases in humans.^3^, ^4^, ^5^ These zoonotic CoVs have claimed thousands of lives in the last 2 decades.^4^ Fortunately, compared to SARS‐CoV and MERS‐CoV, SARS‐CoV‐2 is less pathogenic; however, the concern lies in the fact that it is highly infectious and transmissible. Moreover, asymptomatic Covid‐19 patients have been shown to contribute to the transmission of the infection around the world.^6^, ^7^

Recent reports and studies of SARS‐CoV‐2 in animals have caused unnecessary fear and panic among the population, which have adversely affected the wellbeing and health of animals. Most importantly, it is unethical to cull any animals (wild or domestic) by declaring they are ‘potential’ reservoir hosts without any conclusive evidence that they can transmit SARS‐CoV‐2 to humans. Furthermore, finding the best animal models that mimic human Covid‐19 infection is vital for scientists to understand the pathogenesis of SARS‐CoV‐2 infection. In addition, animal models are critical for testing potential vaccines and therapeutic agents.^8^ A suitable animal model is one that can mimic human disease in terms of viral load, morbidity, clinical signs, and the immune response. Due to the urgency and demand in the current crisis, there is a pressing need to search for a good animal model to control the SARS‐CoV‐2 outbreak.^9^

In this systematic review, we provide a summary of the current evidence on natural and experimental infections of SARS‐CoV‐2 in animals. We compare different animals for their susceptibility, clinical signs, transmission dynamics, and immune response. We also discuss the ability of these animals to mimic Covid‐19 pathogenesis in humans, as well as their usefulness as ideal models for preclinical testing of vaccine candidates and therapeutic agents.

## 2 METHODOLOGY

This systematic review was performed according to the preferred reporting items for systematic reviews and meta‐analyses (PRISMA) statement.^10^

### 2.1 Eligibility criteria

We included published articles that reported infection in domestic or wild animals with serological or molecular confirmation of SARS‐CoV‐2. Inclusion criteria included: (1) investigation of the management of SARS‐CoV‐2 and (2) natural or experimentally induced SARS‐CoV‐2‐infected animal species. Exclusion criteria included (1) infection not clearly defined as SARS‐CoV‐2, (2) non‐animal models, (3) reviews, meta‐analyses, book reviews, letters, abstracts and cases and (4) non‐English reports.

### 2.2 Search strategy

We systematically searched four literature databases (Scopus, PubMed, Science Direct and Web of Science). All databases were searched from December 2019 to October 2020 for relevant studies on Covid‐19 infection in animals. The systematic search was performed using a group of medical subject headings terms and text words, such as: (Covid OR SARS‐CoV‐2) AND (animal OR host OR model OR ‘animal model’ OR Zoonotic OR Pets OR veterinary OR ‘domestic animals’ OR ‘wild animals’ OR ‘animal infection’ OR Mice OR monkey OR Cat OR Dog OR Hamster OR Ferret OR Non‐human primates [NHPs] OR Rabbit OR tiger OR Rodent OR Primate OR Camelidae).

### 2.3 Study selection

**FIGURE 1:**
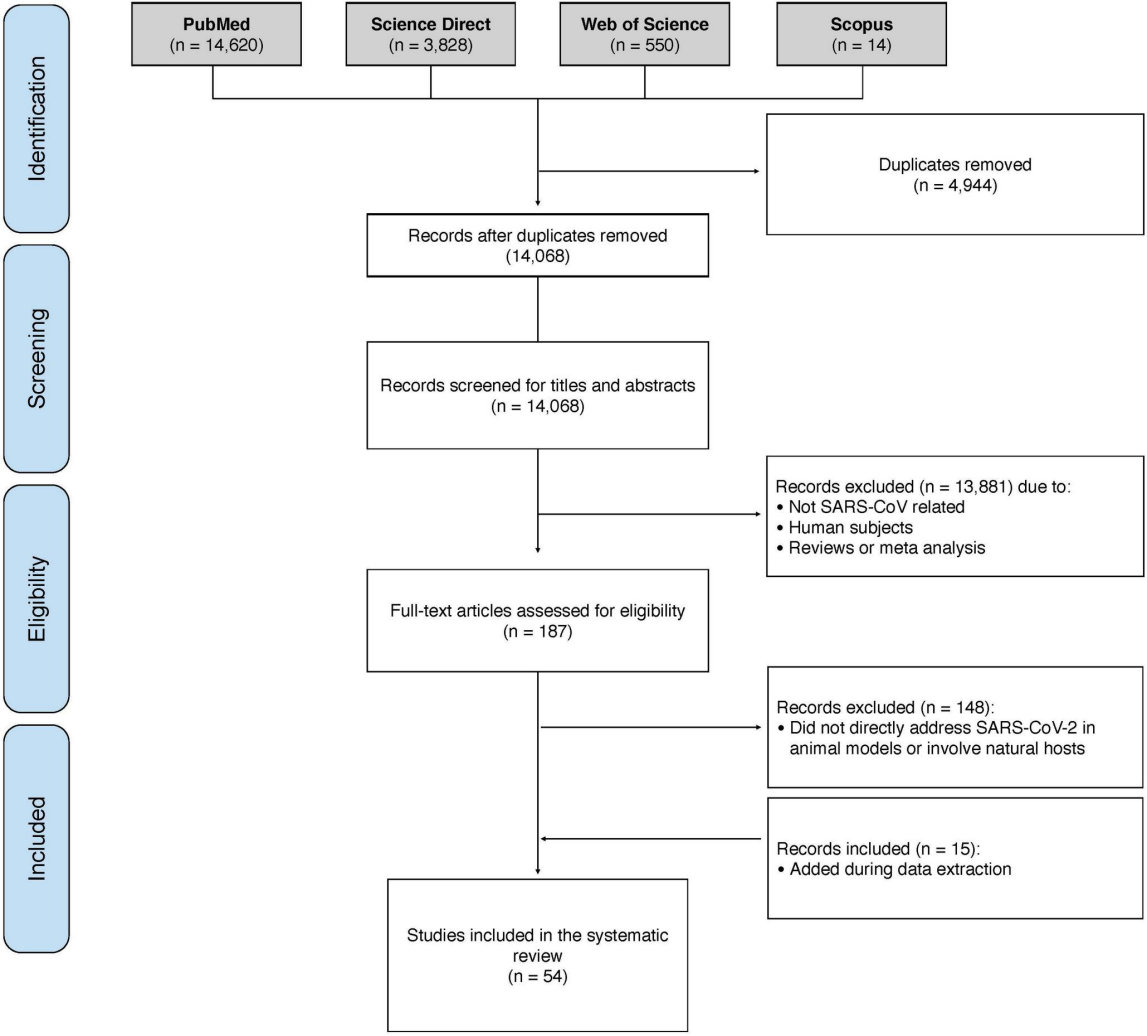
Prisma flow diagram of the systematic review study selection process. The search strategy yielded 19,012 studies. A total of 54 studies were included in the systematic review. SARS‐CoV‐2, severe acute respiratory syndrome coronavirus‐2

**FIGURE 2:**
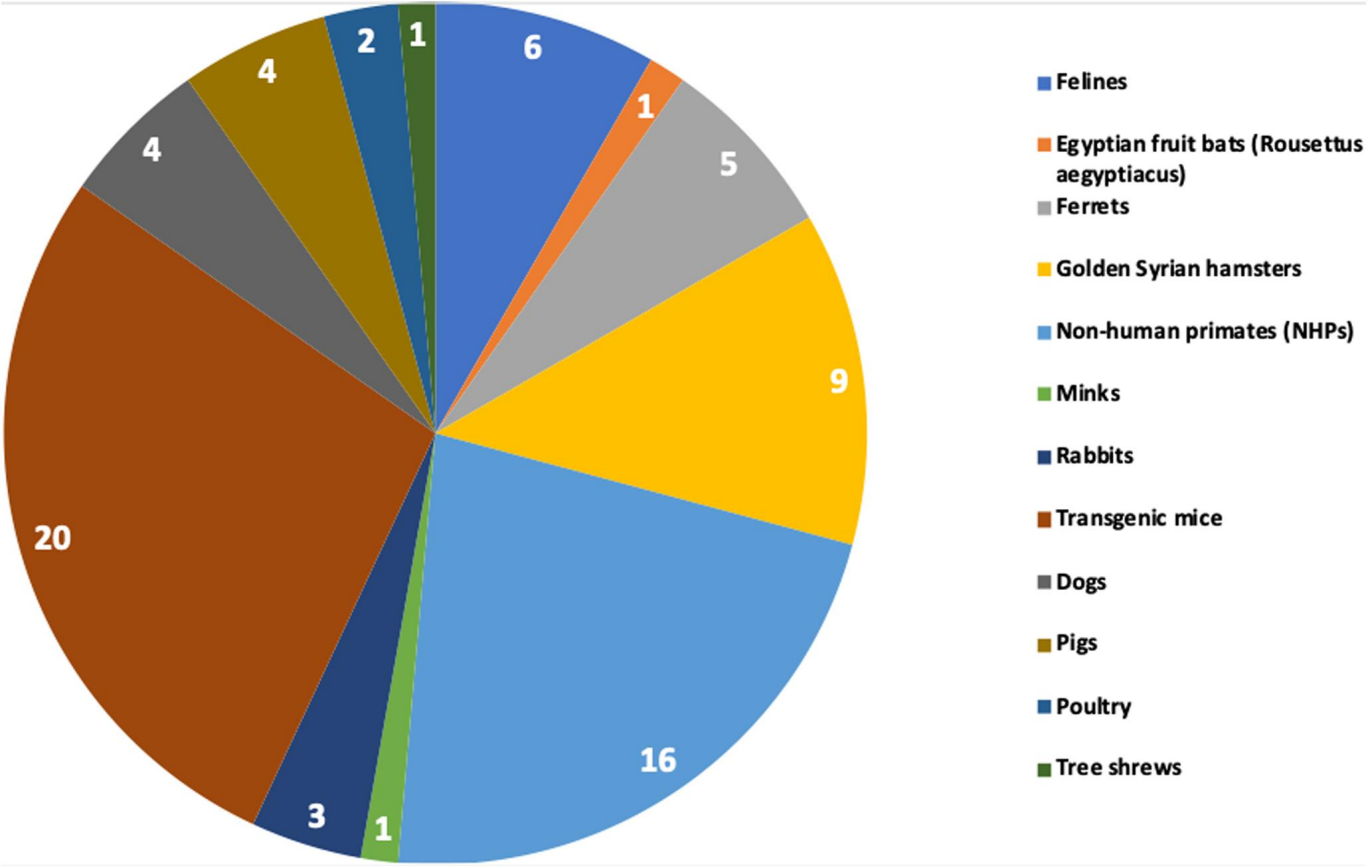
Characteristics of the included studies. Articles obtained from the systematic search were categorized based on the animal used in each study. Most of the studies were conducted on mice and NHPs. Fewer studies were conducted on felines, poultry, dogs, ferrets, fruit bats, minks, hamsters, pigs, rabbits, and tree shrews. Some studies included more than one animal; NHPs, non‐human primates

Four reviewers participated in screening and study selection, of which, three reviewers individually reviewed the identified studies (Salma Younes, Nadin Younes and Farah Shurrab), and a fourth reviewer (Gheyath K. Nasrallah) resolved any discrepancies. All studies retrieved from our initial search strategy were imported to Endnote X9 library, after which, duplicates were removed. Figure 1 illustrates the search strategy. Studies were screened in two stages. The first stage of screening involved screening of titles, abstracts, and keywords. The second stage of screening involved examining the full‐texts of all potentially relevant studies that were identified in the first stage (Figure 1). Any disagreements were resolved by discussion. Bibliographies of the included studies were checked manually for any additional eligible studies not captured in the systematic search Figure 2.

### 2.4 Data collection

Two reviewers (Salma Younes and Nadin Younes) extracted the following data from each included study: animal used, age, number of animals used, type of infection (natural/experimental), inoculation dose, route of challenge, sample, method of detection, immune response, disease and pathology (symptoms), and the possibility of animal‐animal transmission (Table S1). For vaccine and therapeutic studies on animal models infected with SARS‐CoV‐2; the following information were extracted and recorded: animal model, age, number of animals used, inoculation dose, route of challenge, vaccine/antiviral agent, dose, route of inoculation, sample type, method of detection, disease and pathology (symptoms), and the main outcomes of each study (Table S2). The information collected was reviewed independently by all authors to ensure accurate data collection.

**TABLE 1:** Summary of findings of SARS‐CoV‐2 infection in animals

Animals	Type of infection (natural/experimental)	Susceptibility (high/low/none)	Clinical signs	Animal‐animal transmission (Yes/No/NR)	Immune response	Useful as a model (yes/no)	Reference
Felines	Natural and experimental	High	Yes (none to very mild in some cases)	Yes	IgG antibodies detected	No	11, 12, 13, 14, 15, 16
Egyptian fruit bats (*Rousettus aegyptiacus*)	Experimental	High	No	Yes	Neutralizing antibody responses	No	^17^
Ferrets	Experimental	High	No, (very mild in some cases)	Yes	Neutralizing antibody responses	Yes	11, 17, 18, 19, 20
Golden Syrian hamsters	Experimental	High	Yes (none to very mild in some cases)	Yes	Neutralizing antibody responses	Yes	19, 20, 21, 22, 23, 24, 25, 26, 27, 28
Non‐human primates	Experimental	High	Yes	Yes	Antibodies detected	Yes	26, 29, 30, 31, 32, 33, 34, 35, 36, 37, 38, 39, 40, 41, 42, 43, 44
Minks	Natural	High	Yes	Yes	Neutralizing antibody responses	No	^13^
Rabbits	Experimental	High	Yes	N/A	NR	Yes	35, 45, 46
Transgenic mice	Experimental	High	Yes	Yes	Neutralizing antibody responses	Yes	8, 14, 27, 28, 30, 32, 47, 48, 49, 50, 51, 52, 53, 54, 55, 56, 57, 58, 59, 60, 61
Dogs	Natural and experimental	Low	No, (possible in some cases)	No	Neutralizing antibody responses	No	11, 15, 16, 62
Pigs	Experimental	None	No	No	No antibodies detected	No	11, 17, 32, 35
Poultry	Experimental	None	No	No	No antibodies detected	No	11, 17
Tree shrews	Experimental	None	Yes	NR	NR	No	^63^

Abbreviations: NR, not reported; SARS‐CoV‐2, severe acute respiratory syndrome coronavirus‐2.

## 3 RESULTS

### 3.1 Search findings and study characteristics

We identified 19,012 articles, of which 14,068 remained after the removal of duplicates. Thirteen thousand eight hundred eighty‐one irrelevant articles were excluded during the initial stage of screening and 148 articles were excluded during the second stage (Figure 1). The majority of the excluded studies were mainly in vivo human studies with no data on experimental or natural animal species. A total of 54 articles were included in the systematic review.

### 3.2 Animal species and characteristics

The studies collected using the systematic search used various animal species infected with SARS‐CoV‐2 experimentally or naturally. Most of the studies were conducted on mice (37%) and NHPs (30%), fewer studies were conducted on felines, poultry, dogs, ferrets, fruit bats, minks, hamsters, pigs, rabbits, and tree shrews as shown in Figure 2. A consolidative summary of the data gathered from all studies included in this systematic review is given in Table 1. The data collected from each study included in this systematic review are presented in Table S1 and Table S2.

### 3.3 SARS‐CoV‐2 infection in animals

**FIGURE 3:**
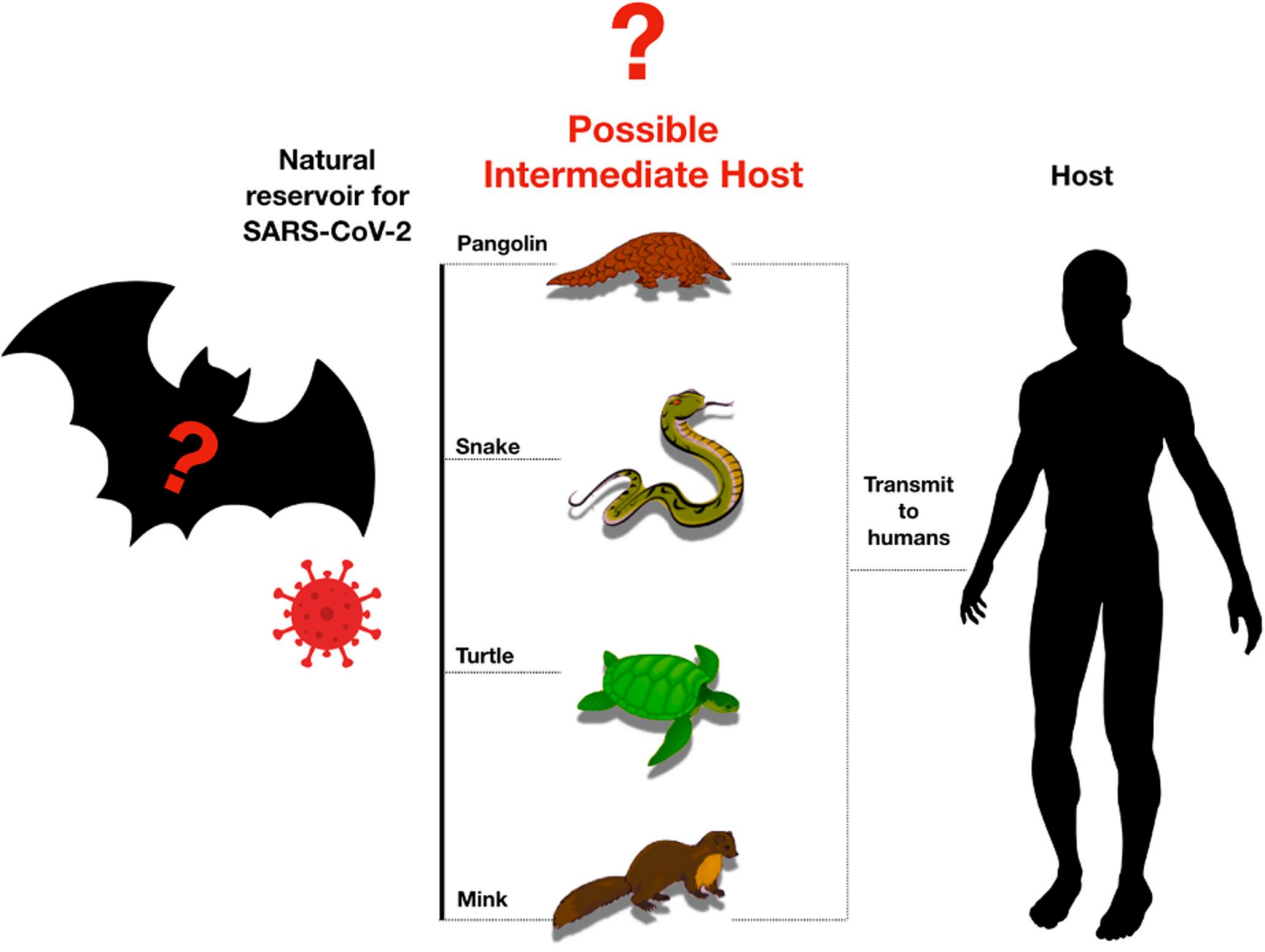
Representative figure of potential intermediate hosts. Bats are the natural reservoir origin of SARS‐CoV‐2. Pangolins are natural hosts of Betacoronaviruses. Metagenomic sequencing identified pangolin‐associated coronaviruses that belong to two sub‐lineages of SARS‐CoV‐2‐related coronaviruses^65^; SARS‐CoV‐2, severe acute respiratory syndrome coronavirus‐2

There is a strong evidence that SARS‐CoV‐2 virus originated in bats^64^; however, the intermediate animal host is still unknown (Figure 3). Several studies showed that pangolins, snakes, turtles, and mink are all possible intermediate hosts, however, further investigations are needed.^66^, ^67^, ^68^ It is worth mentioning that recent findings that analyse the probable animal reservoir to Covid‐19 suggest the snake as a reservoir, based on relative synonymous codon usage bias.^67^ However, the missing link or intermediate link for animal to human transmission of SARS‐CoV‐2 from a recent study suggests the DNA and protein sequence of Malayan pangolins.^69^

**FIGURE 4:**
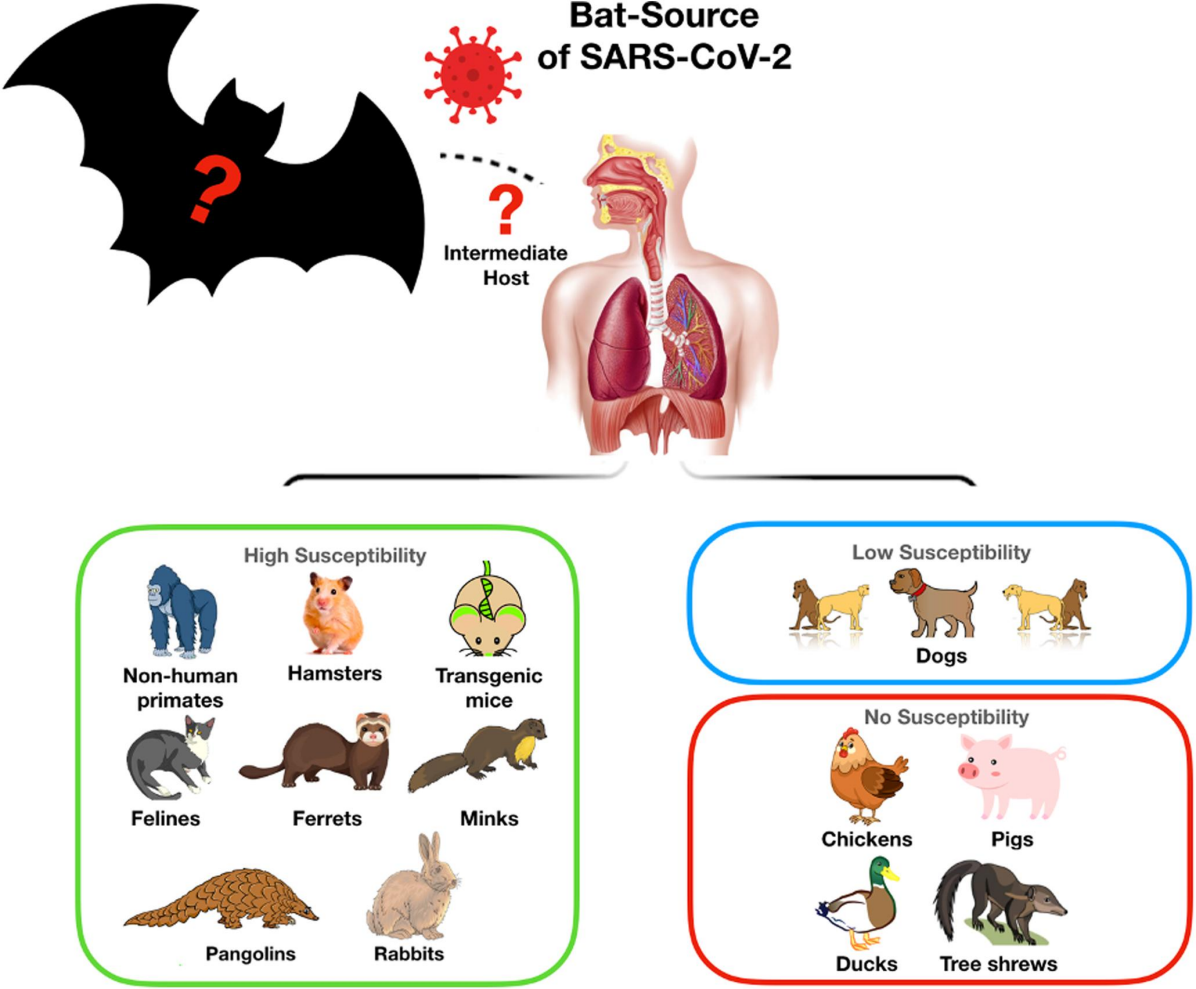
Animal host classification according to susceptibility. NHPs, hamsters, transgenic mice, felines, ferrets, minks, pangolins, and rabbits have high susceptibility, dogs have low susceptibility, while pigs, poultry, and tree shrews have no susceptibility to SARS‐CoV‐2 infection; SARS‐CoV‐2, severe acute respiratory syndrome coronavirus‐2

Studies included in our systematic search revealed low SARS‐CoV‐2 replication in pigs, dogs, chickens, ducks, rabbits and tree‐shrews (Table 1, Table S1 and Figure 4). However, *Rousettus aegyptiacus* (fruit bats), pangolins, felines, mink, ferrets and rabbits were all susceptible to SARS‐CoV‐2 (Table 1, Table S1 and Figure 4). Additionally, tigers, ferrets, mink, macaques, lions, hamsters and cats are susceptible to animal‐to‐animal transmission through the airborne route (Table 1 and Table S1). Moreover, susceptible animals are capable of producing neutralizing antibodies (NAbs) that can offer protection against reinfection (Table S1); however, it remains unclear how long protection will last.

## 4 DISCUSSION

### 4.1 Zoonotic links of SARS‐CoV‐2 outbreak

SARS‐CoV‐2 was first identified in Wuhan city in China in hospitalized patients who previously visited the Huanan wet seafood market where various animals including chickens, pigs, pangolins, bats, snakes, frogs, rabbits, and marmots are sold for human consumption, proposing a suitable environment for zoonotic infection spill‐over to humans.^67^, ^70^, ^71^, ^72^, ^73^ Scientists believe that these traditional Chinese practices might be responsible for the SARS‐CoV‐2 pandemic in humans and that the recurrent interactions between humans and animals without proper biosafety measures present a substantial risk for the occurrence of zoonotic diseases.^74^

Zoonotic CoVs have crossed the species barrier twice in the past 2 decades (SARS‐CoV; 2002 and MERS‐CoV; 2012). Thus, scientists have speculated that SARS‐CoV‐2 resulted from a zoonotic spillover event as well. Zoonotic CoVs need to propagate in their zoonotic reservoirs, and then, seek the chances to spillover via intermediate hosts into susceptible human targets, where they can maintain human‐to‐human transmission. Bats have been revealed as the natural hosts for several human CoVs, including HCoV‐NL63, HCoV‐229E, SARS‐CoV, and MERS‐CoV.^75^, ^76^ Genome sequence analysis confirmed that SARS‐CoV‐2 is 96% identical to the bat CoV RaTG13 at the whole‐genomic level,^77^ and hence bats are believed to be the primary source of origin for the novel SARS‐CoV‐2. However, the intermediate host that is yet to be elucidated.^11^, ^67^, ^70^, ^72^, ^78^ Researchers proposed two hypotheses for the emergence of SARS‐CoV‐2: (1) Natural selection may have occurred in an animal host before transmission to mankind; and (2) natural selection of viruses may have occurred in humans after zoonotic transmission.^79^ In this regard, studies involving the use of animal models or cell culture are in need to help clarify these two scenarios.^79^

### 4.2 SARS‐CoV‐2 infection in animals

Infections with SARS‐CoV‐2 have been documented in various animal species naturally or by experimental infection (Table 1 and Table S1). Our systematic search revealed that *R. aegyptiacus* fruit bats, pangolins, felines, minks, NHPs, hamsters, ferrets and rabbits are all susceptible to SARS‐CoV‐2, dogs are weakly susceptible, whereas pigs, poultry, and tree shrews are not susceptible to SARS‐CoV‐2 infection (Table 1 and Table S1).

#### 4.2.1 Animals that are susceptible to SARS‐CoV‐2

##### Bats

SARS‐CoV and MERS‐CoV originated in bats and were capable of infecting humans.^80^, ^81^ Bats are known to be the ideal hosts for pathogenic CoVs, as viruses remain persistent in asymptomatic bats.^82^ Accordingly, researchers predicted the role of bats as the origin of the current SARS‐CoV‐2 outbreak.^68^, ^82^, ^83^, ^84^ Several studies showed that SARS‐CoV‐2 is virtually identical to the bat CoV on a genomic level, hence it is assumed that bats are the primary source of the SARS‐CoV‐2 spillover.^83^, ^85^ Experimental studies revealed that *R. aegyptiacus* fruit bats are susceptible to SARS‐CoV‐2, as detected by RT‐qPCR, immunohistochemistry, and in situ hybridisation in the nasal cavity of intranasally SARS‐CoV‐2‐inoculated fruit bats^86^ (Table 1 and Table S1).

##### Pangolins

Several studies showed evidence based on metagenomic sequencing analysis that pangolins (*Manis javanica*), a group of endangered mammals, may harbour the ancestral beta‐CoVs, which is the second closest relative SARS‐CoV‐2 virus.^29^, ^65^, ^69^ This CoV identified in pangolin share 85%–92% of their genetic sequence with SARS‐CoV‐2 virus and 90% identity with bat CoV RaTG13 virus. Thus, the pangolin novel CoV cluster into two sub‐lineages of SARS‐CoV‐2‐like viruses in the phylogenetic tree; of which one shares 97.4% amino acid sequence of the receptor binding domain (RBD) with SARS‐CoV‐2.^65^ Until now, the pangolin is probably one of the intermediate hosts of SARS‐CoV‐2.^65^

##### Felines

There have been several reports of human‐to‐feline transmission of SARS‐CoV‐2 (Table 1 and Table S1). Cats from Belgium and Hong Kong tested SARS‐CoV‐2‐ positive.^87^ Cats were shown to be susceptible to SARS‐CoV‐2 and that they may transmit the virus to other cats in close‐contact.^88^ However, none of the infected cats showed symptoms of SARS‐CoV‐2 disease.^11^, ^89^ Recent studies reported that cats develop NAbs against SARS‐CoV‐2, indicating that they can get the infection under natural circumstances and develop an immunological response.^12^ Furthermore, a higher titre of NAbs was observed among cats in close contact with infected owners.^12^ Nevertheless, the extremely low putative infection rates in domestic cats suggest that they are poor hosts for the SARS‐CoV‐2 virus and that the virus is unlikely to become established in wild cats in natural conditions. Other felines, such as tigers and lions have been found to test positive for Covid‐19 after interaction with SARS‐CoV‐2‐infected workers in a Zoo in New York City, USA^90^ (Table 1 and Table S1).

##### Mink

To date, there has been one report of mink infection with SARS‐CoV‐2.^13^ Minks in two mink farms in Beek en Donk and in Milheeze in Netherlands developed signs of breathing and gastrointestinal issues back in April 2020, with a mortality of 1.2% to 2.4%, and deaths mainly observed in pregnant females. Most of the mink showed lung lesions, including interstitial pneumonia.^13^ Investigations later revealed that workers at the farm had previously tested positive for Covid‐19, suggestive of human‐to‐animal transmission (Table 1 and Table S1).

##### Non‐human primates

It has been shown that all NHPs are susceptible to previous SARS‐CoV infection where symptoms of fever, diarrhoea and pneumonitis were reported.^91^, ^92^ Recent studies have shown that rhesus macaques are susceptible to SARS‐CoV‐2 infection. The virus was found to cause mild pneumonia similar to that in humans.^30^ Additionally, infected rhesus macaques developed NAbs that may protect them from subsequent infections.^30^ Other NHPs, such as cynomolgus macaques and common marmosets were shown to be susceptible to SARS‐CoV‐2 infection^29^ (Table 1 and Table S1). However, comparing the susceptibility of rhesus macaques to cynomolgus macaques and common marmosets, the former showed higher susceptibility to SARS‐CoV‐2 infection. It is worth mentioning that viral replication of nasopharyngeal swabs, anal swabs and lung in old monkeys was more active than that in young monkeys for 14 days after SARS‐CoV‐2 challenge.^31^ In addition, old monkeys exhibited diffuse severe interstitial pneumonia compared to young monkeys. Interestingly, a notably higher percentage of lymphocytes was observed in younger monkeys compared to older monkeys, but the decreased counts of lymphocytes were found in both age groups.^31^

##### Hamsters

Several studies have shown that hamsters are susceptible to SARS‐CoV‐2 infection (Table 1 and Table S1). Golden Syrian hamsters infected with SARS‐CoV‐2 developed clinical signs, such as lethargy, ruffled fur, hunched back, abnormal breathing and weight loss.^21^, ^22^, ^23^ In addition, viral transmission to naïve hamsters through direct contact was described.^22^ Of particular interest is the fact that infection with SARS‐CoV‐2 in hamsters reflects some of the demographic differences of Covid‐19 in humans. Thus, aged hamsters and male hamsters seem to develop more severe disease than young and female hamsters, respectively.^24^

##### Ferrets

Ferrets have been described as SARS‐CoV‐2‐susceptible in several studies (Table 1 and Table S1). The virus was shown to replicate efficiently in the upper respiratory tract.^11^ Infected ferrets showed high body temperature and high virus in nasal turbinate, trachea and lungs tissue, accompanied by acute bronchiolitis present in the lungs. Additionally, SARS‐CoV‐2 was detected in saliva and nasal washes of infected ferrets. Furthermore, the virus was transmitted to naïve ferrets that were in close contact with the SARS‐CoV‐2‐infected ferrets.^93^

##### Rabbits

A recent study showed that SARS‐CoV‐2 can infect rabbits, and thus they may serve as potential sources of animal to human transmission (Table 1 and Table S1).^45^ Nevertheless, whether or not rabbits are naturally susceptible to the virus remains under investigation.

#### 4.2.2 Animals that are weakly/not susceptible to SARS‐CoV‐2

##### Dogs

Under natural conditions, there have been few reports of dogs that have tested SARS‐CoV‐2‐positive, of which none reported that dogs could pose as a source of infection for humans (Table 1 and Table S1). So far, studies conducted on dogs that have tested SARS‐CoV‐2‐positive showed that they mostly became infected after close contact with people infected with the virus. In a study conducted in Hong Kong that comprised 15 dogs from households with confirmed SARS‐CoV‐2 cases, only two out of the 15 dogs tested SARS‐CoV‐2‐positive. Both dogs demonstrated antibody responses against SARS‐CoV‐2.^62^ In experimental infection studies, dogs failed to support viral replication and had low susceptibility to the virus,^11^ and thus, failed to show potential for serving as animal models of SARS‐CoV‐2 infection (Table 1 and Table S1).

##### Pigs

A recent study was conducted to investigate whether pigs are susceptible to SARS‐CoV‐2 or not (Table 1 and Table S1). Swabs were found to be free from viral RNA, in addition, no viral RNA was detected in any of the naïve animals in contact, indicating that pigs could not transmit the virus. Pigs used for experimental research, inoculated intravenously, intranasally, ocularly or orally with SARS‐CoV‐2, failed to develop clinical signs. Furthermore, testing sera collected from pigs by enzyme‐linked immunosorbent assay (ELISA) on the 14th day post inoculation (dpi) showed that they were seronegative,^11^ which indicates that pigs did not develop any immune response to the virus. Thus, pigs are unlikely to transmit SARS‐CoV‐2 to humans and are not suitable models for studying SARS‐CoV‐2 infection. These findings are not surprising as previous studies on SARS‐CoV outbreak in 2003 these animals did not play a role as amplifying hosts for SARS‐CoV.^94^

##### Poultry

Recent evidence suggests that poultry are not susceptible to SARS‐CoV‐2 infection^11^ (Table 1 and Table S1). Viral RNA was not detected in swabs taken from SARS‐CoV‐2‐inoculated chickens and ducks. In addition, viral RNA was not detected in any of the naïve animals that were in close contact, indicating no animal‐to‐animal transmission. Furthermore, the inoculated chickens and ducks were found to be seronegative when tested by ELISA.^11^

##### Tree shrews

Experimental infection of tree shrews with SARS‐CoV‐2 has been described^63^ (Table 1 and Table S1). No clinical signs were observed in SARS‐CoV‐2‐inoculated tree shrews except high body temperature,^63^ and thus, may not be a suitable animal for Covid‐19 research.

#### 4.2.3 Potential animal models that are still under research

Other animals are still under investigation whether or not they are susceptible to SARS‐CoV‐2 infection and if they can be useful as models. *In Silico* Analysis revealed that wild felidae, goats, spotted hyenas and civets may be SARS‐CoV‐2‐susceptible and could potentially act as intermediate hosts to transmit the virus from bats to humans.^95^ These findings highlight the usefulness of these animals to be used as experimental animal models for studying SARS‐CoV‐2 infection directly without the need for transgenic operation.^95^ It is noteworthy to mention that rodents, birds, and reptiles are unlikely to be susceptible to SARS‐CoV‐2,^95^ however, further investigation is needed.

### 4.3 Potential animal models to study vaccine and therapeutic candidates for SARS‐CoV‐2 infection

Not every susceptible animal can serve as a good model for testing vaccine and therapeutic candidates. A good animal model is defined by its ability to reproduce relevant human physiology to be used for investigating the effectiveness of potential therapeutic agents and pharmacological drugs. Thus, the identification of reliable and feasible animal models that can mimic Covid‐19 clinical symptoms of humans are urgently needed to decipher the routes of transmission and pathophysiology of the disease. Table S2 summarize the animal models that were utilized for testing vaccines and therapeutic candidates for SARS‐CoV‐2.

#### 4.3.1 Transgenic mice

Mouse models are known to be convenient models to study a disease because they are cheap, available and easy to manipulate. However, these models are challenging to study SARS‐CoV‐2 due to differences in the usage pattern of angiotensin‐converting enzyme 2 (ACE2) receptor compared to human,^96^ which makes them non‐susceptible to the virus, and if they do get infected, it is at a very low rate with poor viral replication. That is because the spike protein of the virus does not recognize the ACE2 receptor of the mouse. However, transgenic mice having human ACE2 (hACE2) receptor supports SARS‐CoV‐2 infection. Several studies utilizing humanized mouse strains expressing hACE2 have been reported^14^, ^47^, ^48^, ^97^ (Table S1 and Table S2). Most of which revealed that hACE2 transgenic mice infected with SARS‐CoV‐2 they can successfully mimic human COVI‐19 symptoms.^14^, ^47^, ^48^ In addition, a study that utilized clustered regularly interspaced short palindromic repeats repetitive/Cas9 knocking technology to generate humanized mouse strains expressing hACE2 revealed that hACE2 mice supported the replication of SARS‐CoV‐2 in pulmonary Clara cells and macrophages,^49^ and they showed symptoms that are similar to those in Covid‐19 patients.^49^ Furthermore, infecting young adult and aged BALB/c mice adapted SARS‐CoV‐2 supported virus replication in both upper and lower airway, with severe symptoms in the aged mice.^48^ Similarly, SARS‐CoV‐2 infection in transducing BALB/c mice encoding hACE2 caused lung pathology, weight loss, and viral pneumonia, where high levels of viral RNA were accumulated in the lungs.^98^ In addition, infecting C57BL/6J (B6J) mice encoding hACE2 with SARS‐CoV‐2 caused pulmonary infiltrates which is similar to Covid‐19 patients.^99^

#### 4.3.2 Ferrets

Ferrets have been widely used to study viral respiratory diseases in humans and remain one of the best candidates as an animal model for respiratory tract infections as they display most of the human‐like symptoms.^71^ Unlike mice, ferrets can be infected with the virus without prior transgenic adaptation. Ferrets were used as animal models for evaluating the effectiveness of antiviral drugs against influenza,^100^ SARS‐CoV^101^ and Ebola.^102^ Thus, researchers believe that ferrets may help in the race for finding the best therapeutic drugs and vaccines against SARS‐CoV‐2. A recent study that investigated the efficacy of different antivirals agents using infected ferrets, has revealed a reduction in the overall clinical scores following treatment with three antiviral agents; lopinavir‐ritonavir, hydroxychloroquine sulphate, and emtricitabine‐tenofovir^18^ (Table S2).

#### 4.3.3 Hamsters

The Syrian hamster is another important animal model to investigate SARS‐CoV‐2 pathogenesis and transmission. The clinical features, histopathological changes, and immune responses reported in infected Syrian hamsters closely mimic those in human Covid‐19 patients.^23^ High replication of the virus was evident in the nasal mucosa and lower respiratory epithelial cells. Interestingly, SARS‐CoV‐2 was observed in the olfactory sensory neurons which parallels the anosmia recorded in Covid‐19 patients.^21^, ^22^, ^23^ NAbs were produced in recovered hamsters, and they had protection against subsequent SARS‐CoV‐2 infection.^21^, ^22^, ^23^ Moreover, passive transfer of serum to naive hamsters was found to efficiently suppress SARS‐CoV‐2 replication in lung tissue^21^ and to provide protection against high dose challenge of the SARS‐CoV‐2 virus,^25^ this occurred without any significant histopathological changes or clinical signs.^23^ Thus, Syrian hamsters are potentially good candidates as a small model for evaluating the efficacy of vaccines and antiviral drugs.^21^ In a study investigating the effect of hydroxychloroquine in hamsters,^26^ no significant difference between infected and control hamsters was observed, indicating that this drug does not inhibit either viral replication or shedding, or further, reduce the clinical symptoms of the disease in hamsters (Table S2).

#### 4.3.4 Non‐human primates

NHPs are considered as one of the best animal models to investigate potential antiviral drugs, therapeutic agents, and to gain a better understanding of the aspects related to pathology in different infectious diseases.^103^ Most of the current knowledge about SARS‐CoV‐2 infection in NHPs is currently preprints, repositories, and inoculation studies. Interestingly, not all infected NHPs showed symptoms of the infection, even though the virus was detected in the nasal swab collections.^104^ These findings resemble the asymptomatic patients, who still can participate in viral transmission without showing any symptoms.^105^ Rosenke and his colleagues tested the effect of hydroxychloroquine on 10 Rhesus macaques infected with SARS‐CoV‐2. Similar to their observations in the hamster model, this drug showed no significant prophylactic or therapeutic benefit in the infected rhesus macaque, similar to humans, despite its promising effects in vitro. Further, six studies were also conducted to test potential therapeutic vaccines and antiviral drugs on NHPs; pigtail macaques,^32^ Rhesus macaque,^26^, ^33^, ^34^, ^35^, ^36^ and cynomolgus macaques^35^ as shown in Table S2. All included studies indicated that NHPs are considered good indicators and preclinical models for testing drugs prior to moving to clinical trials (Table S2).

#### 4.3.5 Other animals

Other animals that have been used for vaccine studies in Covid‐19 research include pigs and rabbits (Table S2). However, there is a lack of studies on these animals, and thus, further investigation on the usefulness of these animals as models is needed.

## 5 LIMITATIONS

This review has several limitations. First, few studies are currently available for inclusion. Second, more detailed information on the collection and sample animals, particularly regarding their clinical findings and conditions during collection, was unavailable in most studies at the time of conducting the review.

## 6 CONCLUSIONS AND FUTURE PERSPECTIVES

This is the first systematic review conducted to summarize current evidence on SARS‐CoV‐2 infection in domestic and wild animals. In summary, SARS‐CoV‐2 has a zoonotic origin and was transmitted to humans via an undetermined intermediate host, leading to infections in humans and other mammals. To enter host cells, the viral spike protein binds to its receptor, ACE2, and is then processed by TMPRSS2. Based on our systematic review, we suggest that SARS‐CoV‐2 can infect a broad range of mammals, but few fish, birds or reptiles. For instance, *R. aegyptiacus* fruit bats, pangolins, felines, minks, NHPs, hamsters, ferrets, and rabbits are all susceptible to SARS‐CoV‐2, and dogs are weakly susceptible. While poultry and pigs, and tree shrews are not susceptible to SARS‐CoV‐2 infection. Mice, mink, hamsters, and ferrets developed clinical signs of the infection that are very similar to that in humans. Thus, these animals might serve as robust useful small‐animal models for future studies to test the efficacy of vaccines and antiviral agents. Whilst, cats, hamsters and ferrets may serve as a useful model for studying the virus transmission and the efficacy of antiviral drugs to limit the spread of the infection. Most importantly, the NHPs were demonstrated to be the most relevant animal model to assess the antiviral agents and vaccine effectiveness before rapid deployment to clinical trials on humans. Although there are more than 180 vaccine candidates and more than 23 well‐known pharmaceutical companies that are trying to produce an effective vaccine against SARS‐CoV‐2, until now, no vaccines were finally approved for human use. However, the current literature data so far proposed that safe vaccines could be available within the coming few months. For more information about vaccine development, the readers are advised to refer back to this comprehensive review and website.^106^, ^107^ It is clear now that among all virus proteins, the most antigenic target for successful active or passive vaccination against all coronavirus, including SARS‐CoV‐2, is the S1 subunit of the spike S proteins; more specifically, the RBDs of the S1 subunit. That is, if a vaccinated host produces NAbs to the RBD, it will block virus binding and, consequently, viral replication. In fact, it was shown that passive vaccination with a cocktail of humanized monoclonal antibodies targeting the RBD has shown effectiveness in the treatment of Covid‐19 patients.^108^ The RBD viral antigen could be injected in a form of self‐replicating mRNA or a recombinant protein. Different delivery systems are currently being used in clinical trials for delivering the virus RNA or the recombinant protein such as the lipid nanoparticles or the non‐pathogenic adenovirus associated vector. Moreover, other companies are also trying to produce live attenuated (a genetically weakened version of the virus) or chemically inactivated whole virus vaccine.^106^ Finally, our systematic search revealed that viral shedding from pets is not sufficient to infect other members of the family or other animals. However, susceptible animals could serve as reservoirs of the virus, necessitating careful ongoing animal management and surveillance.

## CONFLICT OF INTERESTS

The authors declare that they have no competing interests.

## AUTHOR CONTRIBUTIONS

Conceptualization, Gheyath K. Nasrallah; data curation, Salma Younes, Nadin Younes, Farah Shurrab and Gheyath K. Nasrallah; writing—original draft preparation, Salma Younes, Nadin Younes, Farah Shurrab and Gheyath K. Nasrallah; writing—review and editing, Salma Younes, Nadin Younes, Farah Shurrab and Gheyath K. Nasrallah; visualization, Salma Younes, Nadin Younes, Farah Shurrab and Gheyath K. Nasrallah; supervision, Gheyath K. Nasrallah; project administration, Gheyath K. Nasrallah. All authors have read and agreed to the published version of the manuscript.

## Supporting information

Supplementary Material
