# Supplementary material for: Severe acute respiratory syndrome coronavirus‐2 natural animal reservoirs and experimental models: systematic review
Source: Rev Med Virol. 2020 Nov 18;31(4):e2196. doi: 10.1002/rmv.2196 (PMC7744864; doi:10.1002/rmv.2196)
Supplement: Supplementary file 1 — Supplementary Material [file RMV-31-0-s001.docx]

**Table S1.** Studies of SARS-CoV-2 infection in animals.

| Animals | Age | No. of animals | Type of infection (natural/experimental) | Inoculation dose | route of challenge | Viral replication detected in (sample) | Method of detection | Immune response | Disease and pathology (symptoms) | Transmission from animal to animal | Reference | Notes |
| --- | --- | --- | --- | --- | --- | --- | --- | --- | --- | --- | --- | --- |
| Cats | 15–18 weeks-old | 3 | Experimental | 5.2 x 10^5^ PFU | Intranasal, ocular, tracheal, Oral | Nasal and rectal swabs | Virus Titration Assay, ELISA | NR | Virus was detected in all three cats | Yes, Virus titers in the cats that were co-housed with the inoculated cats peaked at 4.5 log10 PFU/ml | (91) | - |
| Cats | 6-9 months | 7 | Experimental | 10^5^ PFU | Intranasal | Fecal sample  Nasal turbinate, soft palates, tonsils, tracheas, lungs, and small intestines | RT-PCR, ELISA, PPNT | Antibodies against SARS-CoV-2 were detected | No signs of infection | Yes: respiratory droplet transmission had occurred | (27) | - |
| Cats | 70-100 days | 10 | Experimental | 10^5^ PFU | Intranasal | Nasal turbinate, soft palates, tonsils, trachea, lungs, and small intestine | RT-PCR, ELISA, PPNT | Antibodies against SARS-CoV-2 were detected | Massive lesions in the nasal and tracheal mucosa epitheliums, and lungs | Yes: the virus is transmissible between cats via the airborne route. | (27) | SARS-CoV-2 can replicate efficiently in cats and that younger cats are more vulnerable than older ones.  One cat died |
| Cats | NR | 102 | Natural | NR | NR | Nasopharyngeal and anal swabs | Indirect ELISA, PPNT, Western blot | 11 had SARS-CoV-2 neutralizing antibodies with a titer ranging from 1/20 to 1/1080 | Asymptomatic | Not done | (40) | 15 of 102 (14.7%) |
| Cats | 6 months to 16 years | 22 | Natural | NR | NR | Serum, nasopharyngeal and rectal swab | RT-qPCR, microsphere immunoassay, and ELISA | 1 cat produced high levels of neutralizing antibodies | 1 cat showed mild respiratory and digestive signs. All the others were negative | Not done | (82) | 1 cat is PCR- positive |
| Cats | NR | 24 | Natural | NR | NR | Serum and oropharyngeal swabs | Microneutralization assay | Seven cats had antibodies against SARS-CoV-2,  detected | No signs of infection; asymptomatic | Not done | (42) | Stray cats in the mink’s farms  Only one cat was positive for viral RNA |
| Cats | NR | 9 | Naturally | NR | NR | Nasal, rectal swabs, and Blood samples | RT-PCR | NR | None of the animals included in this study had been or was infected by SARS-CoV-2 | Not done | (83) |  |
| Chicken | 5 weeks | 20 | Experimental | 10^5^ TCID_50_ | Ocular | Oropharyngeal, cloacal swabs  heart, liver, spleen, duodenum, colon/cecum, pancreas, kidney, adrenal gland, skeletal muscle, skin, and brain | RT-qPCR, next-generation sequencing, antibody detection, histopathology, immunohistochemistry and in-situ hybridization | No antibodies detected | No signs of infection; asymptomatic | Yes: none of contact animals got infected | (84) | Could not be infected intranasally by SARS-CoV-2 |
| Chickens | NR | 3 | Experimental | 10^4.5^ PFU | Intranasal | Oropharyngeal and rectal swabs | RT-PCR, ELISA, | Seronegative for SARS-CoV-2 | None | No transmission | (27) | Not susceptible |
| Dogs | NR | 9 | Naturally | NR | NR | Nasal, rectal swabs, and Blood samples | RT-PCR | NR | None of the animals included in this study had been or was infected by SARS-CoV-2 | Not done | (83) | - |
| Dogs | 2.5 months to 14 year | 11 | Natural | NR | NR | Serum, nasopharyngeal and rectal swab | RT-qPCR, microsphere immunoassay, and ELISA | None | All dogs were negative | Not done | (82) | All dogs were found SARS-CoV-2 PCR negative |
| Dogs | 17 years | 2 dogs positive out of 15 | Natural | NR | NR | Nasal swabs | RT-PCR, PPNT | Neutralising antibodies | Asymptomatic | No transmission | (53) | Acquired the infection from infected owner |
| Dogs | 3 months | 4 | Experimental | 10^5^ PFU | Intranasal | Oropharyngeal and rectal swabs | RT-PCR, ELISA, | Seronegative for SARS-CoV-2 | None | Not done | (27) | 2/4 seroconversion/positive |
| Dogs | 2.5 years | 2 dogs positive out of 15 | Natural | NR | NR | Oral, rectal and nasal swabs | RT-PCR, PPNT | Antibodies detected with titer of 1:80. | Asymptomatic | No transmission | (53) | Acquired the infection from infected owner |
| Ducks | NR | 3 | Experimental | 10^4.5^ PFU | Intranasal | Oropharyngeal and rectal swabs | RT-PCR, ELISA, | Seronegative for SARS-CoV-2 | None | No transmission | (27) | Not susceptible |
| Ferrets | 6 months | 12 | Experimental | 6.10^5^ TCID50  6 hpi, 4 ferrets were co-housed with donor ferret  1 dpi, 4 ferrets were placed in an opposite cage (10 cm) of donor | Intranasal | Throat, nasal and rectal swabs were collected using dry swabs | Real-time RT-qPCR | IgG antibody response against SARS-CoV-2 | NR | Yes: via direct contact and via respiratory droplets between ferrets, 1 to 3 days and 3 to 7 days after exposure respectively | (85) | - |
| Ferrets | 4 months | 6 | Experimental | 5 × 10^4^ PFU | intranasally | Nasal swab | Cytokine response | Reduced interferon type I and III response  Increase proinflammatory chemokines and cytokines response | NR | NR | (86) | - |
| Ferrets | 9-12 month | 12 | Experimental | 10^5^ TCID_50_ | Internasal | Nasal and Rectal swab  Nasal conchae, trachea, lung, tracheobronchial, lymph node, heart, liver, spleen, duodenum, colon, pancreas, kidney, adrenal gland, skeletal muscle, skin, and brain | RT-qPCR, next-generation sequencing, antibody detection, histopathology, immunohistochemistry and in-situ hybridization | Antibodies against SARS-CoV-2 were detected by iIFA in all inoculated ferrets with varying titers (64 to 8192). | Very high replication rate of SARS-CoV-2 in the nasal cavity  None of the ferrets showed clinical signs or loss of body weight during the study period. | Yes: virus was detected in the nasal cavities of contacts | (84) | Virus replication in ferrets resembled similar subclinical symptoms to human with efficient spread. |
| Ferrets | NR | 15 | Experimental | 10^5^ PFU | Intranasal | The nasal turbinate, soft palate, tonsils, trachea, lung, heart, liver, spleen, kidneys, pancreas, small intestine, and brain  For transmission analysis: Nasal washes and rectal swabs | RT-PCR, ELISA, PPNT | Nneutralizing antibody responses | Fever, loss of appetite, lymphoplasmacytic perivasculitis and vasculitis, increased numbers of type II pneumocytes, macrophages, and neutrophils in the alveolar septa and alveolar lumen, and mild peri bronchitis in the lungs. | Yes: SARS-CoV-2 infects the URT of ferrets but is poorly transmissible between individuals | (27) | Two virus strains were used in this study: (i) SARS-CoV-2/F13/environment/2020/Wuhan (F13-E), isolated from an environmental sample collected in the Huanan Seafood Market in Wuhan, and (ii) SARS-CoV-2/CTan/human/2020/Wuhan (CTan-H), isolated from a human patient |
| Fruit Bats (Rousettus aegyptiacus) | NR | 12 | Experimental | 10^5^ TCID_50_ | Internasal | Oral swab and Faecal sample  Nasal conchae, trachea, lung, tracheobronchial lymph node, heart, liver, spleen, duodenum, colon/cecum, pancreas, kidney, adrenal gland, skeletal muscle, skin, and brain | RT-qPCR, next-generation sequencing, antibody detection, histopathology, immunohistochemistry and in-situ hybridization | Neutralizing antibodies could be detected in the same fruit bats with titers up to 64 | Replicated in the URT  No clinical signs such as anorexia or respiratory signs, elevated temperatures, body weight loss or mortality were observed in any of the bats. | Yes: transmitted to contact animals | (84) | Showed characteristics of a reservoir host |
| Syrian hamsters | 6 weeks and 32-34 weeks old | 36 | Experimental | 1 × 10^5^ pfu | Internasal | Blood, nasal washes, bucco-laryngeal swabs, lungs, kidneys, spleens, duodenums, and blood sera were collected | Histopathological examinations and/or virus titrations, RT-qPCR, and serological examination | Young hamsters developed a marked necro-suppurative bronchointerstitial pneumonia with strong alveolar and interstitial influx of neutrophils and macrophages as well as perivascular lymphocytic cuffing, which was much milder or absent in the aged group | Age-dependent SARS-CoV-2-induced body weight losses, with more pronounced weight reductions in aged compared to young hamsters | Not done | (50) | - |
| Golden Syrian hamsters | 4-5 weeks | 9 | Experimental | 8 x 10^4^ TCID_50_ | Intranasal | Nasopharynx aspirate and throat swab.  One lung and one kidney were collected to measure viral load.  Brain, nasal turbinate, lung, liver, kidney, heart, spleen, duodenum and kidney for histopathology | RT-PCR, PPNT, histopathological and immunochemistry examination. | Neutralizing antibodies detected. | Weight loss.  Viral antigens in nasal mucosa, bronchial epithelial cells on 2-5 dpi. Rapid viral clearance and pneumocyte hyperplasia on 7 dpi.  Viral antigen was also found in the duodenum epithelial cells with viral RNA detected in feces. | Yes (transmission via aerosols) | (48) | - |
| Golden Syrian hamsters | 6-10 weeks | Five animals in each group | Experimental | 10^5^ PFU | Intranasal | Nasal turbinate, trachea, lung, extrapulmonary organs (intestine, salivary glands, heart, liver, spleen, lymph nodes, kidney, and brain), and blood | qRT-PCR Histopathology, immunohistochemical, immunofluorescence, TUNEL staining, chemokine/cytokine profiling, and PPNT | All infected hamsters developed serum neutralizing antibody titer ≥1:427 | Clinical signs of lethargy, ruffled furs, hunched back posture, and rapid breathing starting at 2dpi  Histopathological changes from the initial exudative phase of diffused alveolar damage with extensive apoptosis to the later proliferative phase of tissue repair. | Yes: High transmissibility of SARS-CoV-2 among close contact with other hamsters | (49) | - |
| Golden Syrian hamsters | 2 age groups: 1 month; 7-8 months | 8 | Experimental | 10^5.6^, 10^3^ PFU | Intranasal and Ocular | Nasal turbinate, trachea, lungs, eyelids, brain, heart, liver, spleen, kidneys, jejunum, colon, and blood | Micro-CT Imaging and histopathological examination of the lungs,  ELISA, PPNT | Neutralizing antibodies detected. | Severe pathological lung lesions; severe lung injury that shared characteristics with SARS-CoV-2-infected human lungs. | Not done | (47) | Protected against reinfection |
| Golden Syrian hamsters | 6-10 weeks | 52 | Experimental | 10^5^ PFU | Intranasal | Nasal turbinate, trachea and lung | RT-PCR, necropsy, histopathological analysis, PPNT | All challenged index hamsters (n=13) exhibited high titers of serum neutralizing antibodies, ranging from 1:320 to ≥1:640 | Clinical signs of lethargy, ruffled furs, hunched back posture, and rapid breathing starting at 2dpi.  Serological and histological evidence of infection | Yes: Non-contact transmission was found in 66.7% (10/15) of exposed naive hamsters.  Surgical mask partition for challenged index hamsters significantly reduced transmission to only 16.7% (2/12, P=0.019) of exposed naive hamsters.  SARS-CoV-2 could be transmitted by airborne droplet | (49) | - |
| Syrian hamsters  (*STAT2*  *-/-*  and *IL28R-a*  *-/-* ) | 7-12-week-old female STAT2-/- hamsters  5-7-week-old IL28R-a-/- hamsters | 7 | Experimental | 50μl droplets of virus stock containing 2 × 105 TCID50 (P4 virus) or 2 × 106 TCID50 (P6 virus) on both nostrils | Intranasal | Organs were removed and lungs were homogenized, and RNA was extracted | RT-qPCR | High (MMP)-9 levels in lung homogenates compare to WT  Increased IL-6  No increase in serum levels of IL-6, IL-10 and IFNγ | Bronchopneumonia and peribronchiolar inflammation | Not done | (87) | CRISPR/Cas-mediated gene targeting |
| Balb/c mice | 6–8 weeks | 5 | Experimental | 10^5^ pfu | Intraperitoneally | Plasma samples | Microneutralization assay and ELISA | IgG antibody response against SARS-CoV and SARS-CoV-2 spike protein and RBD | NR | Not done | (107) | Cross-reactive antibody binding responses SARS-CoV-2 and SARS-CoV  No cross-neutralization SARS-CoV-2 and SARS-CoV |
| BALB/c mice | 6-10 week | NR | Experimental | 1 × 10^5^ PFU | Intranasally | Serum, bronchoalveolar lavage fluids, lung tissue | RT-qPCR, plaque assay, PPNT, flow cytometry, histology. and immunohistochemistry | Neutralizing antibodies | BALB/c mice lost up to ∼20% of their body weight in the first 4–6 days of infection, and virus grew to high titers in lung tissue and gradually declined over the course of the infection.  Lung tissues demonstrated a variety of lesions including perivascular to interstitial inflammatory cell infiltrates, necrotic cell debris, and alveolar edema | Not done | (8) | Mice are useful for assessing efficacy of vaccines and therapies such as convalescent plasma |
| C57BL/6 mice | 6–8 weeks | 5 | Experimental | 2 × 105 TCID50 | Intranasally | Organs were removed and lungs were homogenized, and RNA was extracted | RT-PCR | Mild inflammatory response | Mild lung pathology | Not done | (87) | - |
| IL28r−/− mice | 6-8 weeks | 5 | Experimental | 2 × 105 TCID50 | Intranasally | Organs were removed and lungs were homogenized, and RNA was extracted | RT-PCR | Mild inflammatory response | Mild lung pathology, | Not done | (87) | - |
| IFNAR−/− mice | NR | NR |  | 1000TCID_50_ | Intranasally | Blood samples and Tissue samples (e.g. lungs, jejunum, liver, heart and kidneys) | Neutralization assay and RT-PCR |  | humped back and erected hairs | Not done | (109) | - |
| C57BL/6 mice | 5-6-month-old | NR | Experimental | 1 × 10^5^ PFU | Intranasally | Serum, bronchoalveolar lavage fluids lung tissue | RT-qPCR, plaque assay, PPNT, flow cytometry, histology. and immunohistochemistry | Neutralizing antibodies | 10%–15% of infected animal suffered weight loss and showed highest virus titers at 1–2 d.p.i.  Lung tissues demonstrated a variety of lesions including perivascular to interstitial inflammatory cell infiltrates, necrotic cell debris, and alveolar edema | Not done | (8) | Mice useful for determining host factors necessary for optimal virus clearance |
| HFH4-hACE2 mice | 8-10 weeks | 34 | Experimental | 7x10^5^ TCID_50_ | Intranasally | Tissues were harvested, including the blood, heart, liver, spleen, lung, brain, kidney, eyes, genital glands, and small intestines. | RT-PCR and Histological analysis | Neutralizing antibodies produced | Mice generated antibodies against SARS-CoV-2 that could neutralize 100 TCID_50_ viruses at dilutions of 1:10 to 1:40.  The infected mice generated typical interstitial pneumonia and pathology that were similar to those of COVID-19 patients.  Viral quantification revealed the lungs as the major site of infection, although viral RNA could also be found in the eye, heart, and brain in some mice | Not done | (58) | SARS-CoV-2 infection localizes to lungs of mice and causes typical interstitial pneumonia  Pre-exposure to SARS-CoV-2 protects mice from exposure |
| Mice | NR | 5 | Experimental | 25 μg recombinant SARS-CoV-2 RBD hFc | Intramuscularly | serum | ELISA | Neutralizing antibodies detected | NR | Not done | (103) | - |
| Transgenic hACE2 mice | 6-11-month-old | 10 | Experimental | 1×10^5^ TCID_50_ | Internasaly | Serum, and different tissues | PCR, ELISA, confocal microscopy, transmission electron microscope and necropsy | IgG antibodies detected | Weight loss and virus replication in lung were observed.  The typical histopathology was interstitial pneumonia with infiltration of significant macrophages and lymphocytes into the alveolar interstitial, and accumulation of macrophages in alveolar cavities. | Not done | (61) | The mouse model with SARS-CoV-2 infection will be valuable for evaluating antiviral therapeutics and vaccines as well as understanding the pathogenesis of COVID-19. |
| Transgenic mice of SARS-CoV-2 expressing hACE2 | Six to twelve-week-old | NR | Experimental | 3x10^7^ PFU/ml | Intranasally | Serum, lung tissues | RT-qPCR, PPNT assay, ELISA, immunohistochemistry | Antibody response detected | None developed significant weight changes or died.  Mild diffuse peribranchial infiltrates | Not done | (98) | Type I interferons are unable to control SARS-CoV2 replication  These mice support viral replication and antibody production and exhibit pathologic findings found in COVID-19 patients |
| Mice (wild type and hACE2 Mice) | 4.5 weeks and 30 weeks | 6 | Experimental | 4 × 10^5^ PFUand 4 × 10^6^ PFU | Intranasally and intragastric gavage | Serum collection and tissue processing. | RT-qPCR, western blotting, histopathological analysis, multiplex immunofluorescent assay, cytokine assay, and immunohistochemistry | NR | None of the inoculated animals displayed obvious clinical symptoms, and only the aged hACE2 mice lost 10% of their weight at 3 dpi and then recovered.  Robust viral RNA replication was seen in lung, trachea, and brain tissues only from both young and aged hACE2 mice | Not done | (62) | High dose of SARS-CoV-2 can establish infection via intragastric route in hACE2 mice |
| Minks | NR | 36 | Natural | NR | NR | Conchae, lung, throat swab and rectal swab | qPCR, next generation sequencing | NR | The symptoms were mostly limited to watery nasal discharge, but some animals showed severe respiratory distress.  Severe diffuse interstitial pneumonia with hyperemia, alveolar damage and loss of air containing alveolar lumina was detected in all harvested lungs. | Not done | (42) | In Netherlands, an increased mink mortality was observed mid-April 2020, which coincide with display of respiratory signs in some animals |
| Pigs | 9 weeks | 12 | Experimental | 105 TCID_50_ | Internasal | Nasal and Rectal swab  nasal conchae, trachea, lung, tracheobronchial lymph node, heart, liver, spleen, duodenum, colon/cecum, pancreas, kidney, adrenal gland, skeletal muscle, skin, and brain | RT-qPCR, next-generation sequencing, antibody detection, histopathology, immunohistochemistry and in-situ hybridization | No antibodies detected | No sign of infection | Yes: none of contact animals got infected | (84) | Pigs could not be infected intranasally by SARS-CoV-2, |
| Pigs | NR | 3 | Experimental | 10^5^ PFU | Intranasal | Oropharyngeal and rectal swabs | RT-PCR, ELISA, | Seronegative for SARS-CoV-2 | None | No transmission | (27) | Not susceptible |
| Rabbits | NR | NR | Experimental | 10^6^ TCID_50_ | NR | Nose, throat, rectum | RT-PCR | NR | No signs of infection; asymptomatic | Not done | (52) | Susceptible |
| Macaca fascicularis | NR | 6 | Experimental | 4.75ml of 106 pfu/ml | Intratracheally, intranasally and conjunctival | Nasal, pharyngeal, and rectal swabs and blood sample | Chest radiography, RT-qPCR, blood biochemical indexes, Virus-specific antibody response, Multiplex analysis of cytokines in serum | Antibodies detected | Increased body temperature was observed in 33.3% (2/6).  Severe gross lesions and histopathological changes were observed in lung, heart and stomach | Not done | (36) | - |
| Callithrix jacchus monkeys | NR | 6 | Experimental | 4.75ml of 106 pfu/ml | Intratracheally, intranasally and conjunctiva | Nasal, pharyngeal, and rectal swabs and blood sample | Chest radiography, RT-qPCR, Blood biochemical indexes, Virus-specific antibody response, Multiplex analysis of cytokines in serum | Antibodies detected | No increase in body temperature.  Severe gross lesions and histopathological changes were observed in lung, heart and stomach | Not done | (36) | - |
| Cynomolgus macaques | 4 - 4.5 years old | 6 | Experimental | 9.13x10^5^ PFU/ml | Direct bilateral primary post-carinal intrabronchial instillation | Blood, conjunctival, nasopharyngeal, oropharyngeal, and rectal swab collection.  Stool and urine were also collected. | Chest CT, Whole-body PET/CT scan, RT-qPCR, ELISA, Serology, Fluorescence neutralization assay, and cytokine analysis | Anti-SARS-CoV-2 IgG antibodiess detected | Mild-to-moderate lung abnormalities | Not done | (90) | - |
| Rhesus macaques | 3-5 years | 5 | Experimental | 1 × 106 TCID50 | conjunctively (CJ), intratracheally (IT), and intragastrically (IG) | Different organ tissues | RT-PCR | IgG anti-SARS-CoV-2 on 21 dpi (CJ route) | Interstitial pneumonia (IT route), Mild interstitial pneumonia (CJ route), No pneumonia (IG route) | Not done | (95) | Infection via the conjunctival route is possible in non-human primates |
| Rhesus macaques | 3-5 years | 4 | Experimental | 10^5^ TCID_50_ | Intratracheal | Nose, pharynx, lung and gut | RT-PCR, ELISA, histopathology, immunohistochemistry, neutralizing antibody assay | Antibodies against S protein detected | Weight loss, reduced appetite,  increased respiration rate, and hunched posture | Not done | (45) | - |
| African green monkeys | NR | 6 | Experimental | 5.0 × 10^5^ PFU | Intratracheal and intranasal routes | Blood, oral swabs, mucosal swabs and bronchoalveolar lavage | Radiographic, histology, RT-qPCR, and plaque titration | Antibodies against N protein detected | No overt clinical signs of disease were observable in any of the animals, other than decreased appetite compared to baseline in 5/6 animals, and a brief period of elevated body temperature suggestive of fever in 2/3 animals,  CRP was elevated two to seven-fold in all animals 2–5 dpi | Not done | (93) | - |
| Rhesus macaque monkeys | 6-12 years old | 9 | Experimental | 1.1 × 10^6^ PFU (Group 1; N = 3), 1.1 × 10^5^ PFU (Group 2; N = 3), or 1.1 × 10^4^ PFU (Group 3; N = 3) | Intranasal and Intratracheal | Bronchoalveolar lavage and nasal swabs | RT-PCR, ELISA, live virus neutralization assay, pseudo virus neutralization assay | All 9 macaques developed binding antibody response by ELISA and Nab by neutralization assay | Animals exhibited modestly decreased appetite and responsiveness suggestive of mild clinical disease as well as mild transient neutropenia and lymphopenia in the high dose group.  Fever, weight loss, respiratory distress, and mortality were not observed. | Not done | (89) | - |
| Rhesus macaque monkeys | 4-6 years | 8 | Experimental | 2.6 x 10^6^ TCID_50_/ml | Intratracheal, intranasal, ocular, and oral | Nasal, throat, rectal swabs, and bronchoalveolar lavages | RT-PCR, Histopathological, immunochemistry examination, transmission electron microscopy, serology, serum cytokine and chemokine analysis | IgG antibodies detected | Irregular respiratory patterns, piloerection, reduce appetite, hunched posture, pale appearance, dehydration, weight loss, pulmonary infiltrates; stress, changes in cytokine and chemokine levels, and prolonged rectal shedding. | Not done | (92) | - |
| Rhesus macaque monkeys | 3-5 years | 4 | Experimental | 1×10^6^ TCID_50_ | Intratracheally re-challenged | Nasopharyngeal and anal swabs | PCR, ELISA, Histopathology and Immunohistochemistry, and neutralizing antibody assay | High levels of neutralizing antibodies | None of the monkeys showed weight loss after re-exposure but the transient elevation of body temperature was observed.  No viral replication in all tissues, as well as no pathological damage and viral antigen in lung tissues. | not done | (61) | Monkeys with SARS-CoV-2 infection after recovery could not be re-infected with the same strain |
| Rhesus macaque monkeys | 3 (3-5 years) & 2 (15 years) | 5 | Experimental | 1 x 10^6^ TCID_50_/ml | Intratracheal | Nasal, throat, rectal swab and lung tissues | RT-PCR, ELISA, Immunohistochemistry, and Haematoxylin and eosin staining | IgG antibodies produced | Weight loss, typical interstitial pneumonia characterized by thickened alveolar septum accompanied with inflammation and edema.  Old monkeys exhibited diffuse severe interstitial pneumonia.  Viral antigens were detected mainly in alveolar epithelial cells and macrophages. | Not done | (46) | - |
| Rhesus macaques | 6–12 years old | 13 | Experimental | NR | Intratracheal and internasal | Bronchoalveolar lavage, EDTA anticoagulated blood, and serum samples | Histopathology, Immunohistochemistry | Innate immune markers increased by SARS-CoV-2 correlated positively with viral loads | Prominent vascular changes including  hyperplastic endothelium and intimal proliferation. Adherence of macrophages and lymphocytes to the endothelium.  Perivascular space  including cytokines and inflammatory markers (IL-6R, IL1RN, IL-10, CXCL10, TNF, NFKB1, and NLRP3), interferon stimulated genes (IRF7, IFIT1, and MX1), IFNα genes (IFNA2, IFNAR2, and IFNA4), and thrombosis-associated markers (C1QA, C3AR1, THBD, and FN1). | Not done | (96) | - |
| Tree shrews | 6–12 Months, 2–4 years, 5–7 years | NR | Experimental | 10^6^ PFU | Intranasal | Nasal, throat, anal swabs and/or blood, lungs, pancreas, uterus | RT-PCR | NR | High body temperature. Pathological alterations in lungs, intestines, spleen, brain, heart, liver, pancreas | Not done | (55) | - |

N/A: Not applicable, NR: Not reported. Dpi = days post-infection, pfu = plaque forming units, TCID50 = mean tissue culture infective dose.

**Table S2.** Studies of SARS-CoV-2 vaccine and therapeutic agents in animal models.

| Animal model | | Age | Number of animals | Challenge dose | Route of challenge | Dose of vaccine/antiviral agent | Vaccine/antiviral agent | | Route of administration | Viral replication detected in (sample) | Method of detection | Disease and pathology (symptoms) | | | Conclusion/outcome | Reference | | |
| --- | --- | --- | --- | --- | --- | --- | --- | --- | --- | --- | --- | --- | --- | --- | --- | --- | --- | --- |
| hACE2 mice | | 10 weeks and 1 year | NR | 105 PFU | Internasally | 2μg | Pegylated-IFN-l1 | | Subcutaneously | Tissue samples | Histopathology and antigen staining | Overt clinical signs of infection (i.e. weight loss) were not observed in young adult BALB/c mice.  High titer virus replication (6.93x10^5^ PFU/tissue) was noted in lung tissue (but was cleared by 4dpi)  High levels of viral antigen | | | mouse-adapted SARS-CoV-2 model demonstrates age-related disease pathogenesis and supports the clinical use of IFN lambda-1a treatment in human COVID-19 infections | (110) | | |
| BALB/c mice | | 8-17 month | 15 | NR | NR | 10 or 1 μg | 10 or 1 μg of LION/repRNA-CoV2S | | Intramuscular injection | Serum | ELISA, Serum chemistries and complete Blood counts | NR | | | Significantly lower antibody titers were observed in the 17-month old mice at both doses, suggesting that higher doses and/or additional booster doses may be required in the most immune senescent populations to induce sufficient immunity | (74) | | |
| BALB/c mice | 12 weeks | | 9 | 1 x 10^5^ PFU | Intranasally | 2μg dose | | Peg-IFN-l1 prophylactically | Subcutaneously | Blood and tissues | qRT-PCR, Histopathology, and neutralization assay | | No overt clinical signs  Decrease in body weight by 3dpi, which was recovered by 4dpi  Old mice showed increased inflammation in the lung at 2dpi and 4dp  High titer virus replication as noted in lung tissue on 2dpi but was cleared by 4dpi | Interferon lambda-1a can potently inhibit SARS-CoV-2 replication in mice.  Mouse-adapted SARS-CoV-2 model demonstrates age-related disease pathogenesis and supports the clinical use of IFN lambda-1a treatment in human COVID-19 infections. | | | (108) | |
| BALB/c mice in expressing hACE2 | | 10-11 weekold | 4-5 mice per group | 4 x 10^5^ PFU | Intranasal | 10 mg/kg, 10 mg/kg, and 5 mg/kg each | mAb COV2-2196, COV2-2130, and COV2-2196 + COV2-2130 | | Intraperitoneal injection | Lung, spleen and hear | Plaque titer of lung tissue, RT-qPCR | Weight loss | | | COV2-2196 or COV2-2130 or their combination prevented severe SARS-CoV-2-induced weight loss  Viral RNA levels were reduced significantly at 7 dpi in the lung and distant sites including the heart and spleen  All animals from COV2-2196 and COV2-2196 + COV2-2130 treatment group and 8 of 10 animals from COV2-2130 treatment, no longer had infectious virus at 2 dpi in the lung.  COV2-2196 or COV2-2130 alone or in combination are promising candidates for treatment or prevention of COVID-19 | (104) | | |
| C57BL/6 mice | | 6-12 week | 15 | NR | NR | N10 or 1 μg | 10 or 1 μg of LION/repRNA-CoV2S | | Intramuscular injection | Spleen and Lungs | ELISA, neutralization assay, serum chemistries and complete Blood Counts | NR | | | 100% seroconversion by 14 dpi and robust anti-S IgG levels with mean binding titers of 200 and 109 μg/ml respectively. | (74) | | |
| Ces1c-/- mice | | 17 week old | NR | 1 x 10^3^ PFU of SARS-CoV-2 RdRp | Intranasally | 25mg/kg | Remdesivir (RDV) | | Subcutaneously or vehicle | Viral load in lung tissue | RT-qPCR and plaque assay | Lung hemorrhage at 5 dpi was significantly reduced with RDV treatment.  RDV significantly ameliorated loss of pulmonary function | | | RDV administration diminished lung viral load and improved pulmonary function as compared to vehicle treated animals.  These data provide evidence that RDV is potently active against SARS-CoV-2 in vitro and in vivo. supporting its further clinical testing. | (99) | | |
| BALB/c | | 6 weeks (n=3) and 9 months (n=3) | 6 | 7.2 × 10^5^ PFU | Intranasally | Mouse-adapted SARS-CoV-2 | None | | None | Lung of each mouse was removed and homogenized | Cytopathic effect, plaque assay, histopathological analysis, cytokine and chemokines analysis, flow cytometry analysis and RT-PCR | Thickened alveolar septa, alveolar damage and focal exudation,  inflammatory cell infiltration, denaturation of endothelial tissues, lung pathology was very similar in old vs. young mice.  Weight loss in the old mice | | | Increased TNF-α, IL-1β, IL-6, and IL-5, MCP-1, G-CSF, and GM-CSF  Higher and sustained cytokines levels in aged mice vs. young | (106) | | |
| Control Mice | | 6-8 weeks | 10 | NR | Intranasally | Challenged with mouse adapted SARS-CoV-2 | Immunized with SARS-CoV-2 RBD-Fc protein | | Intramuscularly | Sera and Lung tissues | Elisa, RT-PCR, and neutralization assay | No abnormalities | | | Higher IgG antibody response against SARS-CoV-2 | (106) | | |
| C3B6: hACE2 mice | | 6-9 weeks | 5 | 10^5^ PFU | Intranasally | SARS-CoV-2 | 0.3 mg of antibody | | Intraperitoneally | Lung tissues | Plaque assay. | No viral replication detectable in lung | | | IgG1 ab1 protects hACE2 transgenic mice from SARS-CoV-2 infection. | (105) | | |
| BALB/c mice | | 6–9 weeks | 6 | 10^5^ PFU | Intranasally | SARS-CoV-2 | None | | None | Lung tissues | Plaque assay. | Viral replication 10^3^ PFU per lung | | | None | (105) | | |
| BALB/c | | 10 weeks and 1 year | 2 | 10^5^ PFU | Intranasally | 10^3^ in 10 μL | Venezuelan equine encephalitis virus strain 3526 based replicon particles (VRPs) expressing SARS-CoV-2 spike (S), nucleocapsid (N), or GFP as control | | Footpad injection | Lungs | Histological analysis, plaque assay, chemokine & cytokine analysis | Decrease in body weight over time | | | VRP-S immunization protecting and significantly limited viral growth and disease severity in the lung of young and aged mice. | (108) | | |
| C57BL/6J | | 10 weeks | 1 | 10^5^ PFU | Intranasally | 10^3^ in 10 μL | Venezuelan equine encephalitis virus strain 3526 based replicon particles (VRPs) expressing SARS-CoV-2 spike (S), nucleocapsid (N), or GFP as control | | Footpad injection | Lungs | Histological analysis, plaque assay, Chemokine & Cytokine analysis | Decrease in body weight over time | | | VRP-S immunization protecting and significantly limited viral growth and disease severity in the lung of young and aged mice. | (108) | | |
| IFNR DKO mice | | 10 weeks | 2 | 10^5^ PFU | Intranasally | 10^3^ in 10 μL | Venezuelan equine encephalitis virus strain 3526 based replicon particles (VRPs) expressing SARS-CoV-2 spike (S), nucleocapsid (N), or GFP as control | | Footpad injection | Lungs | Histological analysis, plaque assay, Chemokine & Cytokine analysis | Decrease in body weight over time | | | VRP-S immunization protecting and significantly limited viral growth and disease severity in the lung of young and aged mice. | (108) | | |
| hACE2 transgenic mice model | | 5-6 weeks old | 12 | 50μL 5x10^5^ TCID_50_ COVID- 19 virus | Intranasally | 25 mg/kg dose | B38 and H4 | | Intraperitoneall | Lung tissues | The virus titer in lungs using qRT-PCR and histopathology of the lungs.  haematoxylin and eosin stain | The body weight of the B38 group decreased slowly and recovered at 3 dpi compared with the phosphate-buffered saline (PBS) control group and the H4 group | | | B38 and H4 are potentially promising virus-targeting monoclonal antibody pair for avoiding immune escape in future clinical applications | (101) | | |
| Mice | | 3-day-old mice | 12 | 10^2^ TCID_50_ | Intranasally | EK1C4 (0.5 mg/kg), EK1 (20 mg/kg) in 2 µl 28% Hydroxypropyl-β-Cyclodextrin (HBC), or phosphate-buffered saline (PBS) solution | EK1C4 and EK1 | | Intranasally | Brain and lungs tissues | Histological examination. | The body weight of mice in the viral control group decreased significantly along with 100% mortality | | | EK1C4 lipopolypeptide could be used for prevention and treatment | (102) | | |
| SPF C57BL/6J (Stock 000664 Jackson Labs) mice | | 20-29 weeks old | 10/dose group | 1 x 10^4^ PFU | Intranasal infection | 50, 150 or 500 mg/kg EIDD-280 | NHC prodrug (EIDD-2801). | | Oral gavage | Lung tissues | Titer of SARS-CoV-2 in lungs by qRT-PCR, plaque assay, whole-body plethysmography, Acute lung injury histological assessment tools | Body weight loss compared to control was significantly diminished.  Lung hemorrhage was also significantly reduced 5 dpi with 500 mg/kg EIDD-2801 treatment.  Dose-dependent reduction in SARS-CoV lung titer. | | | Prophylactic and therapeutic EIDD-2801 significantly reduced lung viral loads and improved pulmonary function and body weight loss in mouse models | (100) | | |
| BALB/c mice | | 11-12 months |  | 10^5^ PFU | Intranasal | different doses | VH-Fc ab8 | | Intraperitoneally | lung was harvested | ELISA, plaque assay, and RT-PCR | NR | | | Bivalent VH, VH-Fc ab8, potently neutralized SARS-CoV-2 in vitro and in animals | (88) | | |
| Ferrets | | 10- 12 months old | 10/treatment group | 10^5.8^ TCID_50_/m | Intranasally | Azathioprine (10 mg/kg), lopinavir (16 mg/kg)-ritonavir (4 mg/kg) (Abbott), hydroxychlo- roquine sulfate (25 mg/kg) (Elyson), or emtricitabine (6 mg/kg)-tenofovir (7.35 mg/kg) | Azathioprine, lopinavir, ritonavir, hydroxychloroquine sulfate, emtricitabine, tenofovir | | oral gavage | Nasal washes and stool specimens  Blood sample was collected  Lung tissues were collected | Neutralizing assay and qRT-PCR | Azathioprine- immunosuppressed ferrets exhibited a longer period of clinical illness, higher virus titers in nasal turbinate, delayed virus clearance, and significantly lower serum neu- tralization antibody titers | | | All antiviral drugs tested marginally reduced the overall clinical scores of infected ferrets but did not significantly affect *in vivo* virus titers | (68) | | |
| Ferrets | | 10-12 months | 10 | 10^5.8^ PFU TCID_50_/ml | Intranasal | 24 mg/kg of body weight/6 mg/kg), | Lopinavir/Itonavir | | Oral gavage | NR | qRT-PCR | Mild fever,4% change in body weight on average | | | Marginally reduced the overall clinical scores of infected ferrets but did not significantly affect  in vivo virus titers | (68) | | |
| Ferrets | | 12-20 months | 10 | 10^5.5^ PFU TCID_50_/ml | Intranasal | 12.5 mg/kg), | Hydroxychloroquine sulfate | | Oral gavage | NR | qRT-PCR | 7% body weight loss | | | Marginally reduced the overall clinical scores of infected ferrets but did not significantly affect in vivo virus titers | (68) | | |
| Ferrets | | 12-20 months | 10 | 10^5.5^ PFU TCID_50_/ml | Intranasal | 6 mg/kg,9 mg/kg | Emtricitabine/Tenofovir | | Oral gavage | NR | qRT-PCR | Mild fever, 4% change in body weight on average and reduced activity | | | Lower virus titers in nasal washes at 8 dpi than the PBS-treated control group | (68) | | |
| Hamsters | | NR | NR | 1 × 10^5^ TCID_50_ | Intranasal | Different doses | VH-Fc ab8 | | Intraperitoneally | lungs were collected | ELISA, plaque assay, and RT-PCR | NR | | | Bivalent VH, VH-Fc ab8, potently neutralized SARS-CoV-2 in vitro and in animals | (88) | | |
| Golden Syrian hamsters | | 6-10 weeks | 30 | 1×10^4^TCID_50_ | Intranasal | 6.5 mg/kg or 50 mg/kg | Hydroxychloroquine | | Intraperitoneal | Oral, rectal swabs and lung tissues | qRT-PCR and necropsy | Ruffled fur, increased respiration rate and reduced mobility  Lungs: focally extensive areas of consolidation that failed to collapse upon removal | | | Prophylaxis or treatment neither the standard human malaria dose (6.5 mg/kg) nor a high dose (50 mg/kg) of HCQ had any beneficial effect on clinical disease or SARS-CoV-2 kinetics (replication/shedding) | (70) | | |
| Golden Syrian hamsters | | 8 weeks old | NR | 1X10^6^ PFU | Intranasal | 16.5 mg/kg | SARS-CoV-2-specific human neutralizing mAb CC12.1 isolated from natural infection | | Intraperitoneally | Viral load in lung tissue | qRT-PCR | NR | | | Multiple highly potent NAbs and passive transfer of nAb provides protection against high- dose SARS-CoV-2.  The study suggests a role for NAbs in prophylaxis, and potential therapy, of COVID-19. | (69) | | |
| Guinea pigs | | NR | 10 | 100 CCID_50_ | NR | A high (8 μg/dose), middle (4 μg/dose), or low (2 μg/dose) | Inactivated Vaccine Candidate: BBIBP-CorV | | Intramuscular and Intravenous | Blood | qRT-PCR, neutralization assay, western blot | No abnormal allergic reactions | | | Neutralizing antibody response detected.  The seroconversion rate reached 100% at 21 days and the NAb levels increased in the high, middle and low dose compared to control.  The anaphylaxis of the positive control group was highly positive (1/6 animals were positive, 3/6 animals were strongly positive, and 2/6 animals were extremely positive) | (77) | | |
| Rabbits | | NR | 8 | NR | NR | 50 μg | Different SARS-CoV-2 spike immunogens | | Intra-muscularly | Sera | ELISA, RBD competition assay, surface plasmon resonance (SPR) against different spike proteins in native conformation, and neutralization assay | NR | | | All three antigens (S1+S2 ectodomain, S1 domain, and RBD), but not S2, generated strong neutralizing antibodies against SARS-CoV-2. RBD immunogen elicited a higher antibody titer with 5-fold higher affinity antibodies to native spike antigens compared with another spike antigen | (97) | | |
| Rabbits | | NR | 10 | 100 CCID_50_ | Intratracheally | A high (8 μg/dose), middle (4 μg/dose), or low (2 μg/dose) | Inactivated Vaccine Candidate: BBIBP-CorV | | Intraperitoneally and intramuscularly | Blood | qRT-PCR, neutralization assay, western blot | NR | | | High levels of neutralizing antibodies | (77) | | |
| Cynomolgus monkeys | | NR | 40 | 100 CCID_50_ | Intratracheally | A high (8 μg/dose), middle (4 μg/dose), or low (2 μg/dose) | Inactivated Vaccine Candidate: BBIBP-CorV | | Intraperitoneally and intramuscularly | Blood | qRT-PCR, neutralization assay, western blot | No cases of death or significant abnormalities in lymphocyte subgroup distribution, cytokines, interferon, interleukin, c-reactive protein, complement, or body weight | | | Neutralizing antibody response detected.  The seroconversion rate reached 100% at 21 days | (77) | | |
| Pigtail macaques | | 3-6 years | 5 | NR | NR | 250 μg dose at week 0 and two macaques received a 50 μg prime at week 0 and a boost at week 4 | 10 or 1 μg of LION/repRNA-CoV2S | | Intramuscularly | Blood | ELISA, Serum Chemistries and Complete Blood Counts | No abnormal weight loss or increase in temperature | | | 100% seroconversion by 14 days post-immunization and robust anti-S IgG levels with mean binding titers of 200 and 109 μg/ml, respectively, and partial seroconversion (2 out of 5) at a 0.1 μg dose | (74) | | |
| Rhesus macaque | | 3-4 years old | NR | 10^6^ TCID50/ml | Intratracheal routes | High dose (6 g/dose), medium dose (3 g/dose | Inactivated SARS-CoV-2 virus vaccine candidate (PiCoVacc) | | Intramuscularly | Lung tissues Blood samples, throat, and anal swabs were collected | Hematological analysis, neutralizing antibody test and RT-PCR | NR | | | Systematic evaluation of PiCoVacc via monitoring clinical signs, hematological and biochemical index, and histophathological analysis in macaques suggests that it is safe | (94) | | |
| Rhesus macaque | | NR | 10 | 2.8 ×10^6^ TCID_50_ | Intratracheal, Oral, Intranasal and Ocular | 6.5mg/kg | Hydroxychloroquine | | Oral gavage | Blood collection, and swabs (oral and nasal) and Bronchoalveolar lavage | qRT-PCR and necropsy | Reduced appetite and ruffled fur followed by pale appearance and irregular increased abdominal respiration | | | HCQ prophylaxis/treatment (6.5 mg/kg) did not significantly benefit clinical outcome nor reduce SARS-CoV-2 replication/shedding in the upper and lower respiratory tract | (70) | | |
| Rhesus macaques | | 6-12 years old | 35 | 1.1 × 10^4^ PFU | Intranasal and intratracheal route | 5 mg DNA vaccines | Wuhan/WIV04/2019  Six versions of S were produced (full length S; deletion of cytoplasmic domain S.dCT; soluble ectodomain S.dTM; S1 domain with fold on trimerization tag S1; receptor binding domain with fold on trimerization tag RBD; soluble ectodomain with deletion of furin cleavage site, PP stabilizing mutations, and fold on trimerization tag S.dTM.PP) | | Intramuscular | Serum, bronchoalveolar lavage and nasal swabs | RT-PCR, plaque assays, ELISA, Pseudovirus neutralization assay, serology, Intracellular cytokine staining assay and western blot | NR | | | Vaccine encoding the full-length S protein resulted in >3.1 and >3.7 log10 reductions in median viral loads in bronchoalveolar lavage and nasal mucosa, respectively.  Vaccine-elicited neutralizing antibody titers  These data demonstrate vaccine protection against SARS-CoV-2 in NHPs. | (78) | | |
| Rhesus macaques | | NR | 12 | 2.6x10^6^ TCID_50_ | Internasal, oral, ocular and intratracheal | 10 mg/kg followed by a daily maintance dose of 5 mg/kg and the other group with 2ml/kg loading dose and 1 ml/kg | Remdesivir | | Intravenous bollus injection | Nasal, throat, rectal swabs and bronchiolar lavage | Quantitative PCR, liquid chromatography mass spectrophotometry and histopathological analysis of all organs | Gross lung lesions were observed in one out of six remdesivir-treated animals.  All six vehicle controls had visible lesions, resulting in statistically significantly difference in the area of the lungs affected by lesions | | | Viral shedding from URT was not reduced by remdesivir, however, at necropsy, lungs viral load of remdesivir-treated animals was lower and there was a reduction in damage to the lungs.  Data support early remdesivir treatment initiation in COVID-19 patients to prevent progression to pneumonia. | (76) | | |
| Rhesus macaques | NR | | 9 | 4 mL intratracheally, 1 mL intranasally, 1 mL orally and 0.5 mL ocularly of 4 x 10^5^ TCID_50_/mL virus suspension | Intratracheally, intranasally, orally, ocularly | 2.5 x 1010 VP/animal diluted in sterile PBS | | ChAdOx1 nCoV-19 virus particles | Intramuscularly | Nasal swabs and blood  Tissues collected: cervical lymph node, mediastinal lymph node, conjunctiva, nasal mucosa, oropharynx, tonsil, trachea, all six lung lobes, right and left bronchus, heart, liver, spleen, kidney, stomach, duodenum, jejunum, ileum, cecum, colon, urinary bladder | Necropsy, neutralization assay, PCR, ELISA Measurement of cytokines and chemokines | | No increase in clinical signs or virus replication throughout the study in vaccinated NHP compared to controls and no markers of disease enhancement in lung tissue of NHPs, such as an influx of neutrophils were observed. | A single vaccination with ChAdOx1 nCoV-19 induced a humoral and cellular immune response in rhesus macaques.  ChAdOx1 significantly reduced viral load in bronchoalveolar lavage fluid and respiratory tract tissue of vaccinated animals challenged with SARS-CoV-2 compared with control animals, and no pneumonia was observed. | | | | (75) |
| Rhesus macaques. | | 3-4 yearold | 6 | l0^6^TCID_50_ | Intratracheally | 2 groups: low-dose (2 μg/dose) or high-dose (8 μg/dose) BBIBP-CorV | Inactivated Vaccine Candidate: BBIBP-CorV | | Intramuscularly | Viral load in lung tissue and viral load in throat and anal swabs | qRT-PCR | The serum biochemical parameters remained constant after vaccination. | | | Neutralizing antibody response detected.  The seroconversion rate reached 100% at 21 days, all macaques in the low- and high-dose groups did not show a detectable viral load in any lung lobe at 7 days after inoculation. | (77) | | |
|  | |  |  |  |  |  |  | |  |  |  |  | | |  |  | | |

N/A: Not applicable, NR: Not reported. Dpi = days post-infection, pfu = plaque forming units, TCID50 = mean tissue culture infective dose.
